# Epigenetic adaptation of the placental serotonin transporter gene (*SLC6A4*) to gestational diabetes mellitus

**DOI:** 10.1371/journal.pone.0179934

**Published:** 2017-06-26

**Authors:** Sofia Blazevic, Marina Horvaticek, Maja Kesic, Peter Zill, Dubravka Hranilovic, Marina Ivanisevic, Gernot Desoye, Jasminka Stefulj

**Affiliations:** 1 Department of Biology, Faculty of Science, University of Zagreb, Zagreb, Croatia; 2 Department of Obstetrics and Gynecology, Clinical Hospital Center Zagreb, Zagreb, Croatia; 3 Division of Molecular Biology, Rudjer Boskovic Institute, Zagreb, Croatia; 4 Department of Psychiatry, Ludwig-Maximilian University of Munich, Munich, Germany; 5 Department of Obstetrics and Gynecology, Medical University of Graz, Graz, Austria; 6 Department of Psychology, Catholic University of Croatia, Zagreb, Croatia; Virgen Macarena University Hospital, School of Medicine, University of Seville, SPAIN

## Abstract

We tested the hypothesis that gestational diabetes mellitus (GDM) alters the DNA methylation pattern of the fetal serotonin transporter gene (*SLC6A4*), and examined the functional relevance of DNA methylation for regulation of the *SLC6A4* expression in the human placenta. The study included 50 mother-infant pairs. Eighteen mothers were diagnosed with GDM and 32 had normal glucose tolerance (NGT). All neonates were of normal birth weight and born at term by planned Cesarean section. DNA and RNA were isolated from samples of tissue collected from the fetal side of the placenta immediately after delivery. DNA methylation was quantified at 7 CpG sites within the *SLC6A4* distal promoter region using PCR amplification of bisulfite treated DNA and subsequent DNA sequencing. *SLC6A4* mRNA levels were measured by reverse transcription—quantitative PCR (RT-qPCR). Functional *SLC6A4* polymorphisms (*5HTTLPR*, *STin2*, *rs25531*) were genotyped using standard PCR-based procedures. Average DNA methylation across the 7 analyzed loci was decreased in the GDM as compared to the NGT group (by 27.1%, p = 0.037) and negatively correlated, before and after adjustment for potential confounder/s, with maternal plasma glucose levels at the 24th to 28th week of gestation (p<0.05). Placental *SLC6A4* mRNA levels were inversely correlated with average DNA methylation (p = 0.010) while no statistically significant association was found with the *SLC6A4* genotypes (p>0.05). The results suggest that DNA methylation of the fetal *SLC6A4* gene is sensitive to the maternal metabolic state in pregnancy. They also indicate a predominant role of epigenetic over genetic mechanisms in the regulation of *SLC6A4* expression in the human placenta. Longitudinal studies in larger cohorts are needed to verify these results and determine to which degree placental *SLC6A4* changes may contribute to long-term outcomes of infants exposed to GDM.

## Introduction

Serotonin (5-hydroxyptamine, 5HT) is a multifunctional signaling molecule, best known for its role in the etiopathogenesis of depression, autism and other mental health conditions [1]. A growing body of evidence points to its contribution also to obesity and related metabolic disorders [2,3]. During embryogenesis, 5HT regulates different developmental processes, including those involved in the development of the serotonergic system itself [4]. Alterations in 5HT homeostasis during the prenatal or early postnatal period, caused by either genetic or environmental influences, affect developmental processes, which may in turn lead to increased disease susceptibility later in life [5,6]. Hence, studying these alterations is of major importance for better understanding of 5HT-related conditions. Before *in situ* expression of 5HT synthesizing enzymes in the fetal brain, the placenta serves as the source of 5HT needed for proper brain development [7]. The placenta is an organ that plays a key role in maintaining homeostasis of the intrauterine environment and is highly responsive to environmental changes [8]. It was thus suggested that the placental 5HT system might be a key link between early environmental perturbations and their impact on developmental outcomes [9,10].

The serotonin transporter (SERT, 5HTT) is an integral membrane protein that plays a central role in the regulation of 5HT homeostasis. SERT mediates uptake of 5HT into cells through high-affinity transport mechanism and is a target of commonly used antidepressant drugs. It is abundant in the brain, where it controls intensity and duration of 5HT synaptic signaling, as well as in several peripheral sites including the human placenta [11]. At the end of a pregnancy, it was found on the villous trophoblast and the endothelium of the feto-placental vessels [12]. Like in other tissues, placental SERT mediates the removal of 5HT, a potent vasoconstrictor, from the extracellular space, thereby contributing to the regulation of uteroplacental blood flow. Human SERT is encoded by a single copy gene located on chromosome 17q12 [13], and also known as the *SLC6A4* (solute carrier family 6 member 4). The *SLC6A4* contains two distinct variable number tandem repeat (VNTR) polymorphisms in the promoter and intron 2 region (*5HTTLPR* and *STin2*, respectively), which, along with promoter single nucleotide polymorphisms (SNPs) *rs25531* and *rs25532*, were shown to modulate transcriptional activity [14]. Increasing evidence implicates also DNA methylation of the *SLC6A4* promoter region in the regulation of *SLC6A4* expression and modulation of human brain function [15–17]. DNA methylation is an epigenetic process through which methyl groups are added to cytosine residues in CpG dinucleotides, thereby modifying gene transcription. Like other epigenetic mechanisms, it is sensitive to environmental influences, particularly during intrauterine development [18]. DNA methylation of the *SLC6A4* promoter region has been shown to associate with several environmental factors including intrauterine exposure to maternal depressed mood [19], perinatal pain-related stress [20], childhood trauma [21,22] and work stress [23].

Intrauterine exposure to maternal hyperglycemia has been repeatedly shown to increase susceptibility for obesity and related metabolic disorders [24–26]. Several recent epidemiological studies have established a link between intrauterine exposure to maternal hyperglycemia and increased risk also for neuropsychiatric and neurodevelopmental disorders including autism [27,28]. Gestational diabetes mellitus (GDM), defined as glucose intolerance with onset or first recognition during pregnancy, is the most common cause of maternal hyperglycemia in pregnancy, affecting a growing number of pregnant women worldwide [29]. Results from human and animal studies suggest that epigenetic modifications of the fetal genome might be central to the molecular mechanisms underpinning long-term consequences of the intrauterine exposure to maternal hyperglycemia [30–34]. Identification of these changes is of great interest given their potential prognostic use [35].

Based on the involvement of 5HT in the regulation of developmental processes, as well as on its pleiotropic roles in mental and metabolic conditions associated with intrauterine exposure to maternal hyperglycemia, we hypothesize that maternal glucose metabolism dysregulation during pregnancy epigenetically affects fetal *SLC6A4* gene, a principal regulator of 5HT homeostasis. Therefore, the present study aimed to investigate potential impact of GDM on the DNA methylation pattern of the fetal *SLC6A4* gene and to examine the functional relevance of DNA methylation for the regulation of *SLC6A4* expression in the human placenta.

## Subjects and methods

### Subjects

The study included 50 mother-infant pairs recruited in the period of six months (from June to November 2015) at the Department of Obstetrics and Gynecology, Clinical Hospital Center Zagreb, Croatia. Demographic, anthropometric and clinical data were extracted from mothers’ and infants’ medical records. In addition, all mothers completed a survey questions about medical history, body measurements as well as medication, vitamins, alcohol and nicotine use before and during pregnancy (S1 Text). These questions have been designed for needs of the present study and have not been validated. GDM diagnosis was based on the International Association of Diabetes and Pregnancy Study Groups (IADPSG) criteria [36] implemented in a Croatian clinical setting [37]. Serum C-reactive protein (CRP) levels were measured using an automated immunoturbidimetric assay on a cobas c 311 analyzer (Roche). Mothers with GDM were treated with an adjusted diet and none was on insulin supplements. In order to minimize the delay between delivery and placental tissue sampling, and to exclude a potential influence of delivery mode on the study results, only singleton pregnancies terminated by elective Cesarean section were considered. Women previously diagnosed with any type of diabetes or having gestational pathologies other than GDM were excluded from the study. Other exclusion criteria were: maternal depression and/or use of serotonergic drugs before or during pregnancy, infant’s birth weight below 2500 g, macrosomia (birth weight above 4500 g [38]), gestational age below 37 weeks, and any known fetal or neonatal abnormalities. Infant's body weight (birth weight) was measured immediately after delivery. Gestational age was the number of weeks from the self-reported first day of the mother's last menstrual period, except for two cases, for which gestational age was corrected on the basis of crown-rump length measured early by ultrasound, because the difference between the two estimates was greater than two weeks. Maternal pre-pregnancy body mass index (pBMI) was computed based on height and pregestational body weight measurements obtained from medical records and confirmed in the questionnaire (cases with inconsistent data were omitted from the study). Gestational weight gain (GWG) was calculated from maternal body weight at delivery and pregestational body weight. Self-reported smoking behavior was dichotomized into 1) never smoking or having quit smoking at least 6 months before the start of pregnancy, and 2) smoking throughout pregnancy or having quit smoking in the first trimester of pregnancy. The study protocol was approved by the Ethics Committee of the Clinical Hospital Center Zagreb and by the Bioethics Committee of the Rudjer Boskovic Institute. Written informed consent was obtained from all women in accordance with the Declaration of Helsinki.

### Placental tissue sampling and nucleic acid isolation

Tissue samples for DNA and RNA isolation were collected from the fetal side of placenta, within 5 minutes after delivery. Decidua was removed and tissue pieces (about 0.5 cm^3^ in size) were excised from 10–12 random positions in each placenta (2–3 positions from each placental quadrant), always avoiding areas of hemorrhage, infarction, calcification or fibrin deposition. Excised tissue pieces were made free of fetal vessels, briefly rinsed in cold physiological saline, and immediately transferred into pre-labelled cryotubes containing preservative solution (RNAlater RNA Stabilization Reagent, Qiagen). Samples were kept at 4°C for 24 h and then stored at -80°C until nucleic acid extraction. Prior to nucleic acid isolation, tissue pieces retrieved from RNAlater were placed on sterile filter paper and an excess of RNAlater solution was removed with an absorbent lab wipe. Genomic DNA was extracted from 15 to 20 mg of tissue using the GenElute Mammalian Genomic DNA Miniprep Kit (Sigma-Aldrich) according to the manufacturer's protocol with optional RNase treatment step. Total RNA was isolated from 10 to 15 mg of tissue using the RNeasy Mini Kit (Qiagen). Optional on-column DNA digestion step (with DNase I, Qiagen) was performed twice to ensure DNA removal. Concentration and purity of isolated DNA and RNA were assessed in triplicates by spectrophotometry (NanoDrop, ND-1000). A_260_/A_280_ values ranged between 1.71 and 1.93 in DNA samples, and between 2.03 and 2.18 in RNA samples; A_260_/A_230_ ratios were above 1.76 in all samples. Aliquots of DNA (500 ng) and RNA (1000 ng) were subjected to agarose gel electrophoresis to verify integrity. All DNA samples produced a single high molecular weight band with no visible smear while all RNA samples displayed sharp 28S and 18S rRNA bands in approximately 2:1 ratio. DNA and RNA samples were stored at -80°C until further processing.

### Genotyping

VNTR polymorphisms in the *SLC6A4* promoter (*5HTTLPR*) and intron 2 (*STin2*) regions were genotyped as described in our previous study [39]. Simultaneous genotyping of *rs25531* (NG_011747.2: 3609A/G) and *5HTTLPR* was based on restriction fragment length polymorphism (RFLP) analysis of PCR products obtained using 50 ng genomic DNA, HotStart Taq DNA Polymerase (Qiagen) and the following primers (0.6 μM) each: forward, 5'-CTCCCTGTACCCCTCCTAGG-3’ (NG_011747.2: 3527–3546) and reverse, 5’-TGCAAGGAGAATGCTGGAG-3' (NG_011747.2: 3801–3819). The cycling conditions were: 95°C for 15 min; 40 cycles of 95°C for 30 s, 60°C for 45 s, 72°C for 45; 72°C for 7 min. PCR products were digested overnight at 37°C with MspI (New England Biolab), electrophoresed using the QIAxcel system, and sized by QIAxcel ScreenGel Software (both from Qiagen). Fragments of 211 and 38 bp corresponded to the S allele, fragments of 245 and 38 bp corresponded to the *La* allele, while fragments of 162, 83 and 38 bp corresponded to the *Lg* allele. The concordance of *5HTTLPR* genotypes obtained by this protocol and the previously mentioned protocol [39] was 100%. In analogy with other studies, *5HTTLPR/rs25531* haplotypes were dichotomized into high-expressing (*La/La*) and low-expressing (other) group [40,41].

### DNA methylation analysis

**Fig 1:**
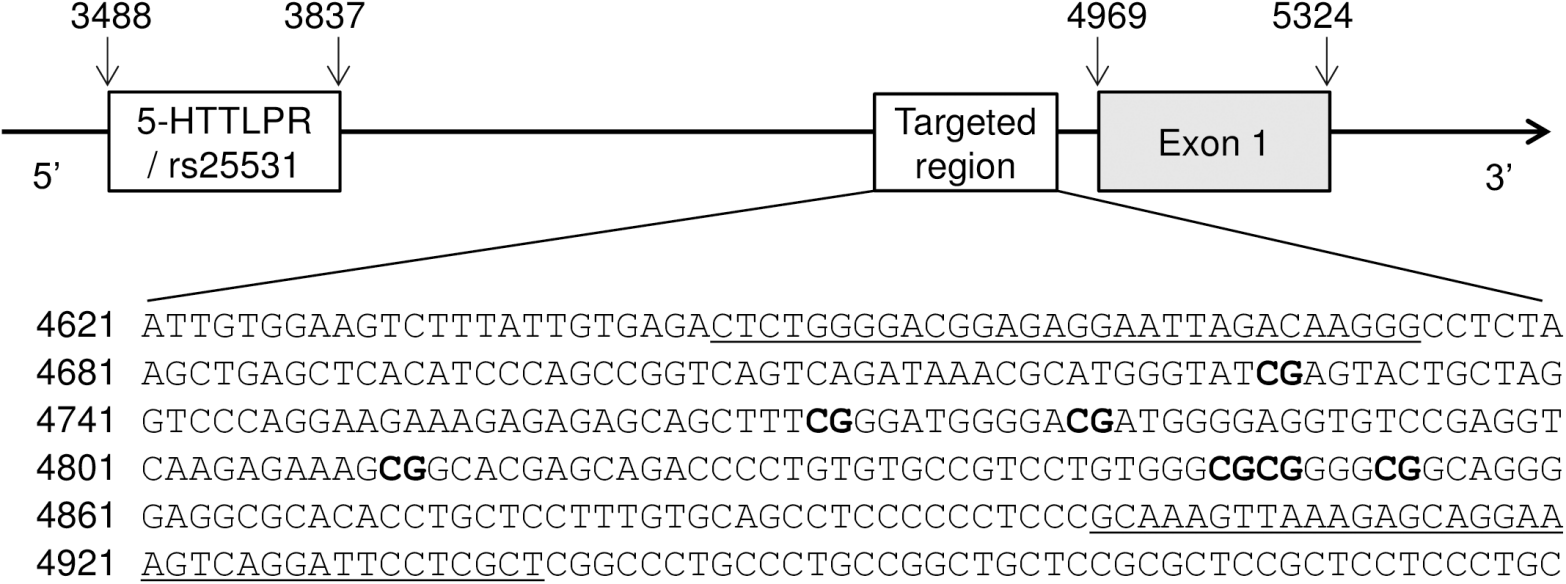
Location and sequence of the *SLC6A4* promoter region targeted by DNA methylation analyses. Numbers indicate nucleotide positions according to NCBI reference sequence NG_011747.2 (GeneBank). Underlined sequences correspond to primers used in PCR. CpG sites found to be methylated in the placental *SLC6A4* gene are shown in bold.

For DNA methylation analysis, we targeted a distal *SLC6A4* promoter region, which has been previously shown to associate with prenatal exposure to maternal depressed mood [19], childhood-related trauma [20,22] and work stress [23]. Fig 1 shows the location of the analysed region in relation to *SLC6A4* exon 1 and *5HTTLPR/rs25531* polymorphism. DNA methylation was quantified using direct bisulfite sequencing [42]. Bisulfite conversion was performed on 750 ng DNA using the EZ-96 DNA Methylation-Gold Kit (Zymo Research). Fully methylated and unmethylated human DNAs were also subjected to bisulfite treatment in order to serve as controls for bisulfite conversion efficiency. Bisulfite-treated DNAs (75 ng) as well as the same amounts of unconverted DNAs were amplified by PCR using HotStart Taq DNA Polymerase (Qiagen) and the following cycling conditions: 95°C for 5 min; 40 cycles of 95°C for 30 s, 56°C for 90 s, 72°C for 2 min; 72°C for 5 min. We tested several sets of primers, designed by the Bisulfite Primer Seeker, in order to choose the one producing the best quality sequence in direct sequencing. The selected primers, yielding a 293 bp amplicon, were: forward, 5'-TTTTGGGGAYGGAGAGGAATTAGATAAGGG (NG_011747.2: 4645–4674), and reverse, 5'-AACRAAAAATCCTAACTTTCCTACTCTTTAACTTTAC (NG_011747.2: 4901–4937). The specificity of PCR products obtained from converted DNAs was confirmed by 2% agarose gel electrophoresis. Control reactions using the original (unconverted) DNA as a template yielded no detectable PCR products. Following purification, PCR products were subjected to bi-directional Sanger sequencing using BigDye chemistry, ABI 3730 capillary sequencer, and Sequencing Analysis software version 5.3.1 (all from Applied Biosystems) as described previously [43,44]. All reactions were run in duplicates and samples marked as unreliable by the Sequence Scanner Software 2 (Applied Biosystems) were discarded. Sequence chromatograms were quantified using the Mutation Surveyor software version 5.0 (Softgenetics) by two independent examiners blind to sample identification codes. Methylation percentage at each CpG site was expressed as the peak height of the cytosine signal relative to the sum of the peak heights of cytosine and thymidine signals [42].

### Gene expression analysis

Relative expression levels of the *SLC6A4* gene were determined by reverse transcription (RT)-quantitative PCR (RT-qPCR) based on SyberGreen detection chemistry. RT was performed with 1500 ng RNA in a final volume of 20 μl using the High Capacity RNA to cDNA Synthesis Kit (Life Technologies) according to the manufacturer’s protocol. cDNA was prepared also from the pool of all RNA samples in order to be used in qPCR validation experiments and as a calibrator sample. Control reactions lacking reverse transcriptase (no-RT) were prepared in order to check for genomic DNA contamination. All cDNAs were diluted to concentration of 10 ng/μl and stored in small aliquots at -20°C. Sequences of primers used in qPCR (S1 Table) were obtained from published literature [45,46]. qPCR assays were prepared with 25 ng of cDNA in a total volume of 20 μl using the Syber Green Master Mix, and run on a 7300 Real Time PCR System (both from Applied Biosystems) according to the manufacturer's recommendations. Each qPCR plate included the calibrator sample and all reactions were run in triplicates. The specificity of the amplicons was verified by melting curve analysis and agarose gel electrophoresis. Experimentally determined qPCR efficiency and optimal working concentration for each primer pair are provided in S1 Table. Control reactions lacking reverse transcriptase (no-RT) yielded undefined quantification cycle (C_q_) in the case of the *SLC6A4* gene, while in the case of other genes, C_q_s obtained from no-RT controls were 14 to 18 cycles higher than that obtained from the respective cDNA samples. Our preliminary analyses including four reference genes identified tyrosine 3-monooxygenase/tryptophan 5-monooxygenase activation protein (*YWHAZ*) as the most stable reference gene according to BestKeeper [47] and Normfinder [48], while ubiquitin C (*UBQ*) followed by *YWHAZ* were the most stable reference genes according to Genorme [49]. These findings accorded with several other studies ranking *YWHAZ* and/or *UBQ* among the most stably expressed genes in human term placenta [46,50–52]. Hence, *SLC6A4* mRNA levels were normalized to mean of *YWHAZ* and *UBQ*. Since qPCR efficiencies of the *SLC6A4* and reference genes were about equal (the slope of log input amount versus ΔC_q_ < 0.1), relative expression levels were calculated using the comparative C_q_ (ΔΔC_q_) method [53].

### Statistical analysis

Bivariate analyses were performed using GraphPad Prism software (version 5.00). The normality of data was assessed by Shapiro-Wilk test. Continuous variables were correlated using Pearson’s (r_p_) or Spearman’s (r_s_) correlation coefficients, as appropriate. Differences between categorical variables were assessed by Student's t-test (with Welch's correction where appropriate), Mann-Whitney U test, one-way analysis of variance (ANOVA), or Kruskal-Wallis test, as appropriate. Chi square (χ^2^) or Fisher's exact tests were used to assess differences in frequency distributions. Interaction between the two independent variables was tested by two-way ANOVA. Partial correlation and multiple regression analyses were performed using IBM SPSS Statistics (version 20) software. All statistical tests were two-tailed and the level of significance was set at p<0.05.

## Results

### Infant and maternal characteristics

Characteristics of infants and their mothers, stratified according to maternal glucose tolerance status, are summarized in Table 1. Eighteen mothers (age range 27 to 42 years) were diagnosed with GDM and 32 mothers (age range 22 to 45 years) had normal glucose tolerance (NGT) according to IADPSG criteria [36]. Numerical results of oral glucose tolerance test (OGTT) performed in the 24th to 28th week of pregnancy were available for 40 participants (results for 10 women with NGT were recorded as "normal"). Serum level of CRP, a biomarker of chronic low-grade inflammation, was measured in 37 participants 1–3 days before delivery. In agreement with previous reports [54], CRP levels were positively correlated with maternal pBMI (r_s_ = 0.397, p = 0.015). Besides elevated plasma glucose concentrations, mothers with GDM had higher pBMI and lower GWG as compared to mothers with NGT, while other sample characteristics did not significantly differ between the GDM and NGT group (Table 1).

**Table 1:** Characteristics of newborns and their mothers stratified according to mother's glucose tolerance status.

Characteristic	GDM (n = 18)	NGT (n = 32)	p-value
NEWBORNS			
Gestational age (weeks)	39. 3 ± 1.4	39.4 ± 1.0	0.79 ^a^
Birth weight (g)	3481 ± 464	3426 ± 390	0.65 ^a^
Sex (males/females, n)	6 / 12	13 / 19	0.76 ^b^
MOTHERS			
Age at delivery (years)	33.8 ± 4.0	32.6 ± 5.3	0.40 ^a^
Parity (primi/multi, n)	7 / 11	17 / 15	0.39 ^b^
pBMI (kg/m^2^)	27.6 (9.7)	22.0 (3.8)	**0.0003** ^c^
Gestational weight gain (kg)	8.8 (7.3)	14.0 (6.8)	**0.0096** ^c^
Smoking in pregnancy (yes/no, n)	6 / 12	7 / 25	0.50 ^b^
Alcohol in pregnancy (yes/no, n) ^d^	3 / 15	5 / 23	0.33 ^b^
Prenatal vitamins (yes/no, n)	16 / 2	26 / 6	0.69 ^b^
Fasting glycemia (mmol/L) ^e^	5.3 ± 0.4	4.6 ± 0.4	**< 0.0001** ^a^
2 hour OGTT glycemia (mmol/L) ^e^	7.3 ± 1.4	5.6 ± 0.9	**0.0002** ^a^
C-reactive protein (mg/L) ^f^	5.1 (5.1)	3.8 (4.7)	0.78 ^c^

Continuous variables are shown as means ± standard deviations or as medians (interquartile ranges). Significant differences between the GDM and NGT group are shown in bold. GDM, gestational diabetes mellitus; n, number of subjects; NGT, normal glucose tolerance; OGTT, oral glucose tolerance test; pBMI, pre-pregnancy body mass inde.

^a^ Student's t-test (with Welch's correction where appropriate)

^b^ Fisher's exact test

^c^ Mann-Whitney U test

^d^ Data for 46 women are shown (4 women with NGT had ambiguous data about alcohol use).

^e^ Data for 40 women are shown (results for 10 women with NGT were recorded as "normal").

^f^ Data for 13 GDM and 24 NGT women are shown.

### DNA methylation status and genotypes of placental *SLC6A4* gene

We identified seven partially methylated CpG cytosines in the targeted *SLC6A4* promoter region, corresponding to nucleotide positions 4728, 4769, 4780, 4811, 4846, 4848 and 4853 in NCBI reference sequence NG_011747.2 (corresponding positions in GRCh38/hg19 are given in S2 Table). CpG cytosines at nucleotide positions 4701, 4717, 4795, 4816, 4835 and 4865 as well as all non-CpG cytosines in the analyzed amplicon were unmethylated yielding only thymidine signals. DNA methylation levels at the seven methylated loci were similar (see S2 Table) and highly correlated with each other (p<0.0001 for all combinations, mean r_s_ = 0.81; for individual correlation coefficients see S3 Table). Hence, average DNA methylation across the seven CpG sites (referred to as *SLC6A4* methylation) was calculated for each placental sample and used in further analyses as previously applied for blood cell DNA methylation of the respective *SLC6A4* region [16,21,22,41].

In order to consider the possible influence of adjacent genetic variants on *SLC6A4* methylation levels, all placental samples were genotyped for known functional polymorphisms in the *SLC6A4* promoter (*5HTTLPR*, *5HTTLPR/rs25531*) and intron 2 (*STin2*) regions. The observed genotype frequencies (S4 Table) accorded with Hardy-Weinberg equilibrium (p = 0.468, 0.471 and 0.104 for *5HTTLPR*, *5HTTLPR/rs25531* and *STin2*, respectively; χ^2^ test). *SLC6A4* methylation levels did not differ as a function of these polymorphisms analyzed either separately or in combinations (p = 0.327–0.828).

### Association of *SLC6A4* methylation with infant and maternal characteristics

**Fig 2:**
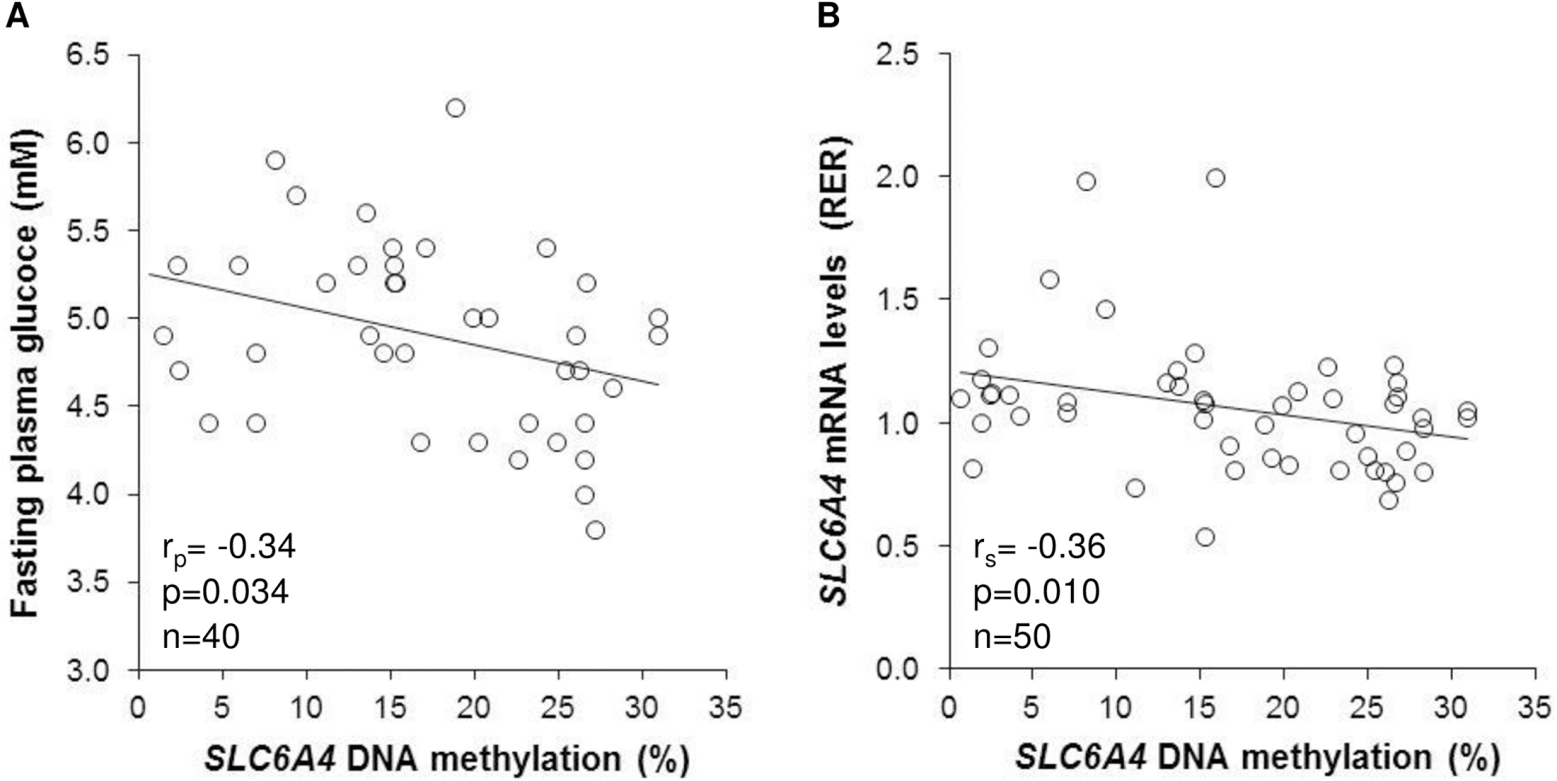
Placental *SLC6A4* methylation levels correlate with maternal fasting glucose concentrations and placental *SLC6A4* mRNA levels. Scatterplots depict correlation of placental *SLC6A4* methylation with (A) maternal fasting glucose levels in the 24th to 28th week of pregnancy, and (B) placental *SLC6A4* mRNA levels. n, number of subjects; r_p_, Pearson's correlation coefficient; r_s_, Spearman's correlation coefficient; RER, relative expression ratio.

*SLC6A4* methylation was decreased in placentas of infants born to mothers with GDM as compared to infants of mothers with NGT (by 27.1%) while no statistically significant associations were found with infant sex, parity, maternal body weight status, and nicotine, alcohol or vitamins use (Table 2). Further, there were no statistically significant correlations between *SLC6A4* methylation levels and gestational age (r_s_ = 0.22, p = 0.126), birth weight (r_s_ = 0.21, p = 0.147), maternal age at delivery (r_s_ = -0.21, p = 0.145), pBMI (r_s_ = 0.01, p = 0.945), GWG (r_s_ = 0.05, p = 0.7504) or circulating CRP levels (r_s_ = 0.004, p = 0.98). On the other hand, *SLC6A4* methylation levels were negatively correlated with maternal fasting plasma glucose concentrations in the 24th to 28th week of pregnancy (Fig 2A). The correlation remained statistically significant after adjusting for potentially confounding variable/s (S5 Table) including maternal pBMI (p = 0.009 and 0.022 for fasting and 2 h OGTT glucose concentration, respectively) and GWG (p = 0.028 and 0.034 for fasting and 2 h OGTT glucose concentration, respectively).

**Table 2:** Association of placental *SLC6A4* methylation with categorical variables of the study population.

	n	*SLC6A4* methylation (%)		p-value
		mean	sd	
**Infant sex**				
males	19	18.5	9.6	0.318 ^a^
females	31	15.7	9.3	
**Maternal glucose tolerance**				
GDM	18	13.5	6.4	**0.037** ^a^
NGT	32	18.6	10.4	
**Pre-pregnancy body weight** ^c^				
normal	30	16.9	10.2	0.665 ^b^
overweight	10	16.0	8.8	
obese	10	18.1	8.2	
**Parity**				
0	24	18.4	10.0	0.137 ^a^
≥1	26	15.2	8.8	
**Smoking in pregnancy**				
no	37	17.1	9.6	0.466 ^a^
yes	13	15.6	9.0	
**Alcohol in pregnancy**				
no	38	16.3	9.6	0.805 ^a^
yes	8	15.7	9.9	
**Prenatal vitamins use**				
no	8	19.8	8.2	0.296 ^a^
yes	42	16.2	9.6	

Significant differences between categories are shown in bold. n, number of subjects; NGT, normal glucose tolerance; GDM, gestational diabetes mellitus; pBMI, pre-pregnancy body mass index; sd, standard deviation.

^a^ Mann-Whitney U test

^b^ Kruskal-Wallis test

^c^ Normal weight, overweight and obesity were defined as pBMI<25, 25≤pBMI<30 and pBMI≥30, respectively

### Placental *SLC6A4* mRNA levels

**Fig 3:**
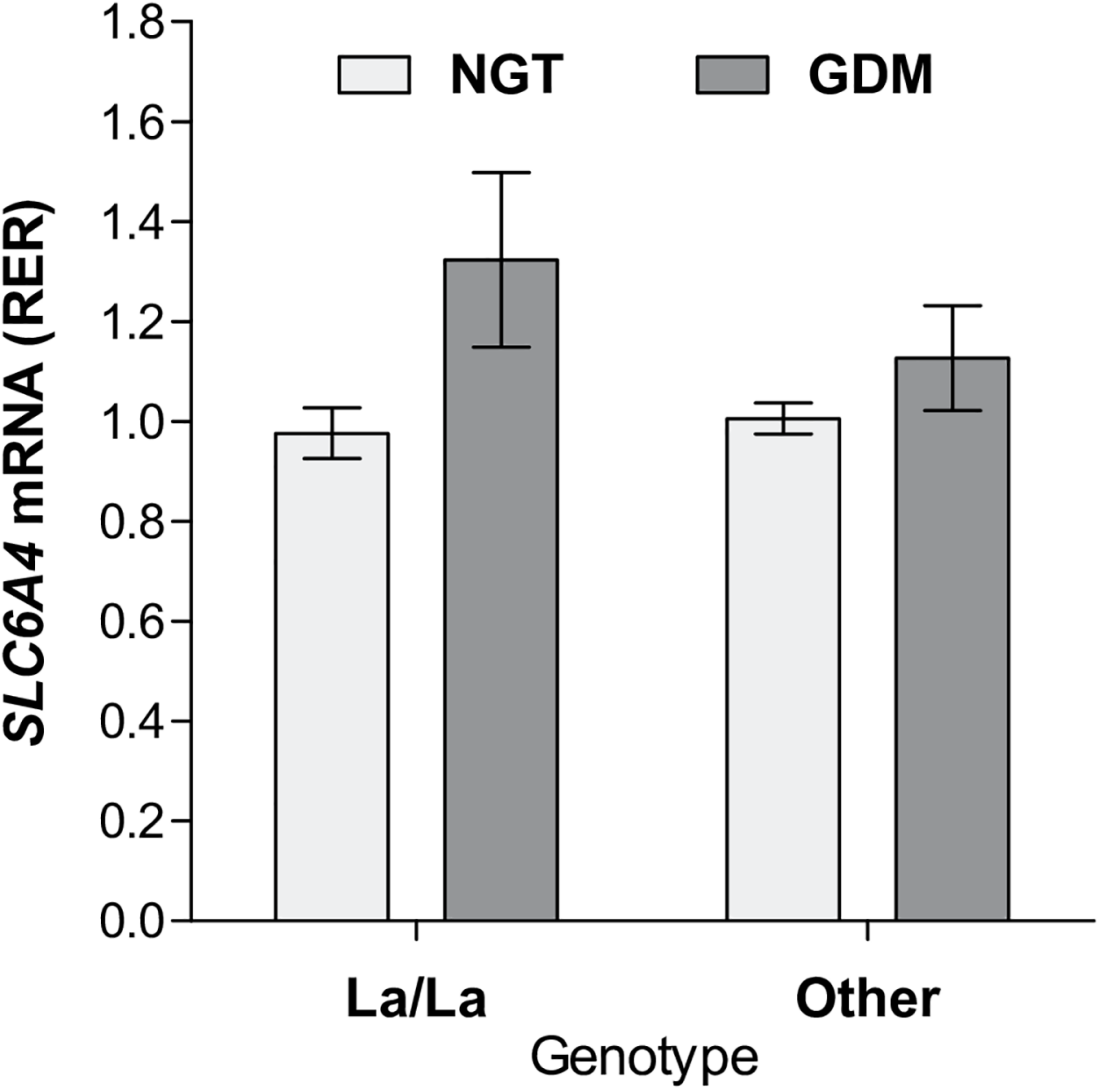
Placental *SLC6A4* mRNA levels according to maternal glucose tolerance status and *5-HTTLPR*/*rs25531* genotype. Shown are means and standard errors. p-values were 0.007, 0.316, and 0.176 for the main effect of diagnosis, main effect of genotype, and genotype x diagnosis interaction, respectively (two-way ANOVA). GDM, gestational diabetes mellitus; NGT, normal glucose tolerance; RER; relative expression ratio.

To investigate whether the *SLC6A4* methylation marks associated with GDM have functional relevance, we quantified *SLC6A4* mRNA levels in the respective placental samples. A statistically significant negative correlation was found between *SLC6A4* methylation and placental *SLC6A4* mRNA levels (Fig 2B). Two-way ANOVA revealed a significant effect of diagnosis (p = 0.007), while there were no statistically significant effects of *5HTTLPR/rs25531* genotype and diagnosis x genotype interaction on placental *SLC6A4* mRNA levels (Fig 3). Infant sex, gestational age, maternal age at delivery, parity, pBMI, GWG, serum CRP levels, and nicotine, alcohol, or the use of prenatal vitamins were all unrelated to placental *SLC6A4* mRNA levels (p>0.05 in all cases), while a negative correlation was observed between placental *SLC6A4* mRNA levels and infant's birth weight (r_s_ = -0.36, p = 0.011).

## Discussion

The present study is the first to address the potential impact of maternal metabolic state in pregnancy on the epigenotype of fetal 5HT-related genes. In particular, we investigated the relationship between GDM, the most common cause of maternal hyperglycemia in pregnancy, and placental DNA methylation of the *SLC6A4* gene. We found significantly decreased *SLC6A4* methylation in placentas of newborns exposed to GDM as compared to newborns from the NGT group (Table 2). This finding suggests that epigenetic marks at the fetal *SLC6A4* loci are responsive to maternal metabolic derangements associated with GDM. Indeed, we found a negative correlation between *SLC6A4* methylation and maternal glucose concentrations in the 24th to 28th week of pregnancy (Fig 2A), which remained statistically significant after adjusting for potential confounders including pBMI and GWG (S5 Table). Although the correlation between *SLC6A4* methylation and maternal pBMI was not statistically significant (see Results section), the contribution of maternal obesity, which is a major risk factor for GDM [55], to the epigenetic modifications of the fetal *SLC6A4* gene could still be biologically important and should be more thoroughly explored on larger samples.

We found inverse correlation between placental *SLC6A4* methylation and *SLC6A4* mRNA levels (Fig 2B). This finding, being in line with the generally observed decreasing effect of promoter-associated DNA methylation on transcriptional activity [56], strongly supports a functional role of the CpG sites studied here in the regulation of placental *SLC6A4* gene expression. As shown on Fig 3, we found no statistically significant association between placental *SLC6A4* mRNA levels and *5HTTLPR/rs25531* genotypes. Additional analyses of potential effects of *5HTTLPR/rs25531*, *5HTTLPR* and *STin2* polymorphisms on placental *SLC6A4* mRNA levels, assuming either a complete or partial dominance model, or combining the *5HTTLPR* and *STin2* polymorphisms as in our previous study on lymphoblasts [57], also yielded no statistically significant results (p-values ranged between 0.615 and 0.963). Collectively, these findings suggest a predominant role of epigenetic over genetic mechanisms in regulating *SLC6A4* gene expression in the human placenta. This notion resonates with the placenta's inherent capability to adapt to various environmental changes [58]. Several other layers of gene regulation such as histone modifications and non-coding RNAs can be also modified by environmental cues. Their potential contribution to regulating placental *SLC6A4* gene expression remains to be studied in the future.

We have performed an exploratory analysis of possible effects of DNA methylation at individual CpG sites. Association of GDM (S1 Fig) as well as maternal fasting plasma glucose concentrations in pregnancy (S6 Table) with DNA methylation was most pronounced for the most proximal loci 4846, 4848 and 4853. The same sites, along with the 4811 locus, showed statistically significant negative correlation with placental *SLC6A4* mRNA levels (S6 Table). Interestingly, peripheral blood DNA methylation at CpG sites 4846 and 4848 has been shown to correlate with *in vivo* measures of human brain 5HT synthesis [16] while peripheral blood DNA methylation at CpG site 4853 was identified to confer the strongest association with clinical response to antidepressant treatment with selective serotonin reuptake inhibitors (SSRI) [41]. Furthermore, cord blood DNA methylation at sites 4846 and 4811, along with a few other sites, has been associated with prenatal exposure to maternal depressed mood [19]. Taken together, CpG sites 4846, 4848 and 4853 seem to be particularly relevant for the regulation of *SLC6A4* function and should come in focus of future epigenetic studies of this gene.

Placental *SLC6A4* hypomethylation in the GDM group along with the inverse correlation between placental *SLC6A4* methylation and mRNA levels infer an up-regulation of placental *SLC6A4* mRNA levels in GDM. As shown in Fig 3, GDM was indeed associated with a significant up-regulation of placental *SLC6A4* mRNA levels. Although our results differ from the study reporting decreased *SLC6A4* mRNA levels in placentas obtained from GDM as compared to non-GDM pregnancies [12], they are in line with an *in vitro* study showing that diabetes-like concentrations of glucose increase transcription of the *SLC6A4* gene in human placental choriocarcinoma (JAR) cells [58]. A possible explanation for the discrepancy between the *in vivo* findings may relate to the use of different reference genes for normalization, the modality of GDM management (i.e. diet and insulin [12] versus solely diet in our study), diagnostic criteria, mode of delivery, tissue sampling procedure, gestational weight gain and/or other subject characteristics that differed between the two studies. The functional consequences of up-regulation of placental *SLC6A4* mRNA levels in GDM remain to be identified, especially in light of the reported GDM-related decrease in 5HT uptake rate [59, 60, 61].

Interestingly, we have observed a statistically significant (p = 0.011) negative correlation between placental *SLC6A4* mRNA levels and infant's birth weight. We subsequently conducted multiple linear regression analysis with birth weight as the outcome and placental *SLC6A4* mRNA levels along with several covariates (in particular, infants sex and gestational age, maternal smoking in pregnancy, glucose tolerance status and GWG) as predictors (S7 Table). The model statistically significantly predicted birth weight in our sample of normal birth weight infants (F_7,42_ = 4.958, p<0.000, R^2^ = 0.45, R^2^_adjusted_ = 0.36) and *SLC6A4* mRNA levels added significantly to the prediction (β = -0.311, p = 0.018). It remains to be studied whether the placental *SLC6A4* gene might be involved in the regulation of fetal growth by including neonates with a birth weight at both extremes of the birth weight range.

Our study has several strengths. First, it was performed in a well-defined cohort of subjects recruited from the same geographical area that is inhabited by an ethnically homogenous population of southern Slavic (prevalently Croatian) descent. All deliveries were by Cesarean section, ruling out potential effects of vaginal delivery and labor. Because of the close correlation of the degree of methylation at the seven sites analysed, we calculated the average methylation across the region as a more robust measure than methylation levels at individual loci. Also, functional *SLC6A4* polymorphisms were determined to account for their possible effects on methylation frequency and mRNA levels. The weakness of the study, besides its relatively small sample size, is the analysis of total placental tissue instead of its cell components. This is the case with all other epigenetic studies of the placenta in GDM. Although we cannot rule out that the cellular composition of the placenta samples was different between the GDM and control cases, affecting thereby the epigenetic results [62], the mild forms of GDM studied here make it unlikely. We have not measured SERT protein levels in our samples, but it was reported earlier that its transcript and protein levels correlate [12].

In conclusion, results of the present study suggest that GDM-related glucose alterations in maternal blood influence DNA methylation of the fetal *SLC6A4* gene and that DNA methylation plays a more important role than *SLC6A4* polymorphisms in the regulation of *SLC6A4* expression in the human placenta. We also found that *SLC6A4* mRNA levels in placenta were associated with birth weight. Studies in larger and independent cohorts are needed to confirm these findings and to determine whether and to which degree other GDM-related parameters such as maternal pBMI and its associated biochemical alterations (circulating cytokines, blood lipid profiles, etc.) influence placental *SLC6A4* methylation and expression. The recently demonstrated cross-tissue convergence of *SLC6A4* methylation [15] and the correlation between the blood and brain methylomes [63] may suggest that the placental changes found here could reflect fetal/neonatal *SLC6A4* brain methylation. This certainly warrants further studies testing potential associations of placental *SLC6A4* changes with long term outcome of the neonates.

## Supporting information

S1 Text The survey questions used in the study, in the original (Croatian) and English language. (PDF)

S1 Table Gene-specific primers used in real-time PCR (qPCR) analyses. (PDF)

S2 Table Genomic coordinates of the methylated CpG sites in placental *SLC6A4* promoter region and the observed methylation frequencies in the overall sample. (PDF)

S3 Table Spearman’s correlation coefficients between placental DNA methylation levels at the individual CpG sites in the *SLC6A4* promoter region. (PDF)

S4 Table Frequencies of *5HTTLPR*, *5HTTLPR/rs25531* and *STin2* genotypes in the overall sample. (PDF)

S5 Table Partial correlation between the *SLC6A4* methylation levels and maternal plasma glucose concentrations in the 24th to 28th week of pregnancy. (PDF)

S6 Table Correlation of placental DNA methylation levels at individual CpG sites in the *SLC6A4* promoter region with maternal fasting plasma glucose levels in the 24th to 28th week of pregnancy, and with *SLC6A4* mRNA levels in the human placenta. (PDF)

S7 Table Linear regression analysis for predicting infant's birth weight. (PDF)

S1 Fig Placental DNA methylation at individual *SLC6A4* loci in infants of mothers with normal glucose tolerance and mothers with gestational diabetes mellitus. (PDF)
