# Supplementary material for: Epigenetic adaptation of the placental serotonin transporter gene (SLC6A4) to gestational diabetes mellitus
Source: PLoS One. 2017 Jun 26;12(6):e0179934. doi: 10.1371/journal.pone.0179934 (PMC5484502; doi:10.1371/journal.pone.0179934)
Supplement: S1 Text — (PDF) [file pone.0179934.s001.pdf]

1. Datum popunjavanja: ____

2. U kojem ste tjednu trudnoće? _______________________________________

3. Vaša dob (godine): ____

4. Vaše tjelesne mjere:

Visina u cm:______________

Sadašnja težina u kg:______________

Težina u kg prije ove trudnoće (zadnji menstrualni ciklus):_____________

5. Molimo Vas, zaokružite lijekove/vitaminske pripravke koje ste uzimali **tijekom ove trudnoće**. Ako znate, navedite naziv lijeka/pripravka.

a) pripravci folne kiseline ____

b) vitaminski pripravci za trudnice (npr. Prenatal, Elevit) ____________________________

c) lijekovi za opuštanje (npr. Normabel, Xanax) ______________________________________

d) lijekovi protiv depresije (npr. Prozac, Zoloft) ______________________________________

e) neki drugi lijek/vitaminski pripravak____

f) nisam uzimala nikakve lijekove ni vitaminske pripravke

6. Jeste li **prije ove trudnoće** uzimali koji od gore navedenih lijekova ili vitaminskih pripravaka?

a) ne

b) da   Ako da, koji? ____

Prije koliko vremena? ____

7. Jeste li **tijekom ove trudnoće** patili od navedenih poremećaja/bolesti? Ako da, zaokružite.

a) dijabetes	e) depresivni poremećaj	g) epilepsija
b) povišeni krvni tlak	f) bipolarni poremećaj	i) migrena
c) problemi sa štitnjačom	g) anoreksija ili bulimija	j) ovisnost o alkoholu
d) anksiozni poremećaj	h) shizofrenija	k) ništa od navedenog

l) neki drugi poremećaj/bolest (navedite): ____

8. Jeste li bilo kad **prije ove trudnoće** patili od gore navedenih poremećaja:

a) ne

b) da   Ako da, od kojeg? ____

9. Da li trenutno pušite duhanske proizvode?

a) ne, nikad u životu nisam pušila

b) ne, prestala sam pušiti prije više od godinu dana

c) ne, prestala sam pušiti kad sam saznala da sam trudna

d) da, trenuno pušim

Ako trenutno ne pušite, odgovorite na pitanje **10** i nastavite s pitanjem **12**.

Ako trenutno pušite, odgovorite na pitanje **11** i nastavite s pitanjem **12**.

10. Jeste li ikad u životu pušili?
a) ne
b) da   Ako da, kad ste prestali (godina, mjesec)?______________________________________
Koliko ste prosječno cigareta dnevno pušili? __________________________________

11. Koliko cigareta dnevno popušite?

Prije trudnoće:
a) 30 ili više
b) 20 do 30
c) 10 do 20
d) manje od 10
Prosječan broj:_______________

Tijekom ove trudnoće:
a) 30 ili više
b) 20 do 30
c) 10 do 20
d) manje od 10
Prosječan broj:_______________

12. Koliko često pijete alkohol?

Prije trudnoće:
a) svaki dan
b) 5 ili 6 puta tjedno
c) 3 ili 4 puta tjedno
d) 1 ili 2 puta tjedno
e) rjeđe nego 1 puta tjedno
f) rjeđe nego 1 puta mjesečno
g) nikad

Tijekom ove trudnoće:
a) svaki dan
b) 5 ili 6 puta tjedno
c) 3 ili 4 puta tjedno
d) 1 ili 2 puta tjedno
e) rjeđe nego 1 puta tjedno
f) rjeđe nego 1 puta mjesečno
g) nikad

13. Koliki broj alkoholnih pića (*) prosječno popijete u prilikama kad pijete alkohol?

Prije trudnoće:
a) 9 ili više
b) 5 do 8
c) 3 ili 4
d) 1 ili 2
e) nikad ne pijem

Tijekom ove trudnoće:
a) 9 ili više
b) 5 do 8
c) 3 ili 4
d) 1 ili 2
e) nikad ne pijem

14. Koliko često popijete 5 ili više alkoholnih pića (*) u jednoj prilici?

Prije trudnoće:
a) češće nego jednom tjedno
b) otprilike jednom tjedno
c) otprilike jednom mjesečno
d) rjeđe nego jednom mjesečno
e) nikad

Tijekom ove trudnoće:
a) češće nego jednom tjedno
b) otprilike jednom tjedno
c) otprilike jednom mjesečno
d) rjeđe nego jednom mjesečno
e) nikad

***** jedno alkoholno piće = 1 standardno pivo (5 dcl)
1-2 dcl vina
oko 0.5 dcl likera
oko 0.3 dcl žestokog pića

1. Filling date: ____

2. What week of pregnancy are you in? ____

3. Your age (years): ____

4. Your body measurements:

Body height in cm: _______________

Current body weight in kg: _______________

Body weight in kg before this pregnancy (last menstrual cycle): ________________

5. Please circle the medication or vitamins that you have been taking **during this pregnancy**.
If you know, please write the name of the medication / vitamin supplement.

a) folic acid supplements ____

b) prenatal multivitamins (e.g. Prenatal, Elevit) ______________________________________

c) anti-anxiety drugs (e.g. Normabel, Xanax) ____

d) antidepressants (e.g. Prozac, Zoloft) ____

e) other medications/vitamin supplements ____

f) I have not been taking any medication or vitamin supplements

6. Have you taken any of the above mentioned drugs or vitamins ever **before this pregnancy**?

a) no

b) yes   If yes, which? ____

How long ago?____

7. Have you suffered from any of the mentioned disorders/diseases **during this pregnancy**?
If yes, please circle the corresponding ones:

a) diabetes	e) depressive disorder	g) epilepsy
b) high blood pressure	f) bipolar disorder	i) migraine
c) thyroid problems	g) anorexia or bulimia	j) alcohol dependence
d) anxiety disorders	h) schizophrenia	k) none of the mentioned

l) other disorder/disease (please specify): ____

8. Have you suffered from any of the above mentioned disorders ever **before this pregnancy**?

a) no

b) yes   If yes, from which? ____

9. Do you currently smoke tobacco?

a) no, I have never smoked in my life

b) no, I stopped smoking more than a year ago

d) no, I stopped smoking when I found out I was pregnant.

e) yes, I currently smoke

If you currently don't smoke, answer the question **10** and proceed with question **12**.

If you currently smoke, answer the question **11** and proceed with question **12**.

10. Have you ever smoked in the past?

a) no

b) yes If yes, when did you quit (year, month)? ______________________________________

How many cigarets on average have you smoked per day? _______________________

11. How many cigarettes do you smok per day?

<u>Before pregnancy</u>:
a) 30 or more
b) 20 to 30
c) 10 to 20
d) less than 10
Average number:________

<u>During this pregnancy</u>:
a) 30 or more
b) 20 to 30
c) 10 to 20
d) less than 10
Average number:________

12. How often do you consume alcohol?

<u>Before pregnancy</u>:
a) every day
b) 5 or 6 times per week
c) 3 or 4 times per week
d) 1 or 2 times per week
e) less than once per week
f) less than once per month
g) never

<u>During this pregnancy</u>:
a) every day
b) 5 or 6 times per week
c) 3 or 4 times per week
d) 1 or 2 times per week
e) less than once per week
f) less than once per month
g) never

13. How many drinks (*) do you usually have on occasions when you consume alcohol?

<u>Before pregnancy</u>:
a) 9 or more
b) 5 to 8
c) 3 or 4
d) 1 or 2
e) I never drink

<u>During this pregnancy</u>:
a) 9 or more
b) 5 to 8
c) 3 or 4
d) 1 or 2
e) I never drink

14. How often do you have 5 or more drinks (*) on one occasion?

<u>Before pregnancy</u>:
a) more often than once a week
b) about once a week
c) about once a month
d) less than once a month
e) never

<u>During this pregnancy</u>:
a) more often than once a week
b) about once a week
c) about once a month
d) less than once a month
e) never

***** one drink = 1 beer (5 dl)
1-2 dl wine
about 0.5 dl of light liquor
about 0.3 dl of hard liquor
