# Supplementary material for: Epigenetic adaptation of the placental serotonin transporter gene (SLC6A4) to gestational diabetes mellitus
Source: PLoS One. 2017 Jun 26;12(6):e0179934. doi: 10.1371/journal.pone.0179934 (PMC5484502; doi:10.1371/journal.pone.0179934)
Supplement: S1 Table — (PDF) [file pone.0179934.s002.pdf]

S1 Table. Gene-specific primers used in real-time PCR (qPCR) analyses.

Name of gene	Symbol	Primer sequence (5' - 3')	Concentration (μM)	qPCR efficiency[a]	Coefficient of regression[b]	Sequence source
Serotonin transporter	*SLC6A4*	f: TGGTTCTATGGCATCACTCAGTTC<br>r: GTTGTGGCGGGCTCATCAG	0.40	96.3%	0.997	[1]
Tyrosine 3-monooxygenase / tryptophan 5-mono-oxygenase activation protein, zeta polypeptide	*YWHAZ*	f: CCGTTACTTGGCTGAGGTTG<br>r: AGTTAAGGGCCAGACCCAGT	0.20	97.5%	0.998	[2]
Ubiquitin C	*UBQ*	f: TCGCAGCCGGGATTTG<br>r: GCATTGTCAAGTGACGATCACA	0.40	96.6%	0.995	[2]

f, forward; r, reverse.

[a]qPCR efficiency and [b]coefficient of regression were calculated from slopes of 6-point standard curves with two-fold serial dilutions.

References

1. Van Lelyveld N, Ter Linde J, Schipper MEI, Samsom M. Regional differences in expression of TPH-1, SERT, 5-HT3 and 5-HT4 receptors in the human stomach and duodenum. Neurogastroenterol Motil. 2007;19: 342–348. doi:10.1111/j.1365-2982.2006.00891.x
2. Baumann M, Körner M, Huang X, Wenger F, Surbek D, Albrecht C. Placental ABCA1 and ABCG1 expression in gestational disease: Pre-eclampsia affects ABCA1 levels in syncytiotrophoblasts. Placenta. 2013;34: 1079–1086. doi:10.1016/j.placenta.2013.06.309
