# Supplementary material for: Epigenetic adaptation of the placental serotonin transporter gene (SLC6A4) to gestational diabetes mellitus
Source: PLoS One. 2017 Jun 26;12(6):e0179934. doi: 10.1371/journal.pone.0179934 (PMC5484502; doi:10.1371/journal.pone.0179934)
Supplement: S2 Table — (PDF) [file pone.0179934.s003.pdf]

S2 Table. Genomic coordinates of the methylated CpG sites in placental *SLC6A4* promoter region and the observed methylation frequencies in the overall sample (n=50).

NG_011747.2	GRCh38/hg19 (chr17)	Frequency of methylated cytosines (%)				
		Min	Median	Max	Mean	sd
4728	30.236.209	0.00	15.8	32.4	15.6	9.8
4769	30.236.168	0.00	17.3	27.5	17.0	7.6
4780	30.236.157	0.00	16.8	29.9	15.0	9.8
4811	30.236.126	0.00	16.2	35.1	15.8	12.5
4846	30.236.091	0.00	17.1	35.2	17.1	11.5
4848	30.236.089	0.00	19.0	33.9	19.7	9.0
4853	30.236.084	0.00	17.8	34.7	17.1	12.1

Min, minimum; Max, maximum; sd, standard deviation.
