# Supplementary material for: Epigenetic adaptation of the placental serotonin transporter gene (SLC6A4) to gestational diabetes mellitus
Source: PLoS One. 2017 Jun 26;12(6):e0179934. doi: 10.1371/journal.pone.0179934 (PMC5484502; doi:10.1371/journal.pone.0179934)
Supplement: S3 Table — (PDF) [file pone.0179934.s004.pdf]

S3 Table. Spearman's correlation coefficients between placental DNA methylation levels at the individual CpG sites in the *SLC6A4* promoter region. n=50, p<0.0001 for all combinations.

**CpG** [a]	**4728**	**4769**	**4780**	**4811**	**4846**	**4848**	**4853**
**4728**							
**4769**	0.79						
**4780**	0.77	0.87					
**4811**	0.78	0.76	0.71				
**4846**	0.89	0.83	0.81	0.87			
**4848**	0.90	0.83	0.78	0.88	0.96		
**4853**	0.77	0.72	0.72	0.69	0.81	0.83	

[a] Cytosine position according to NCBI reference sequence NG_011747.2 (GeneBank)
