# Supplementary material for: Epigenetic adaptation of the placental serotonin transporter gene (SLC6A4) to gestational diabetes mellitus
Source: PLoS One. 2017 Jun 26;12(6):e0179934. doi: 10.1371/journal.pone.0179934 (PMC5484502; doi:10.1371/journal.pone.0179934)
Supplement: S4 Table — (PDF) [file pone.0179934.s005.pdf]

S4 Table. Frequencies of the *5HTTLPR*, *5HTTLPR/rs25531* and *STin2* genotypes in the overall sample (n=50).

**Polymorphism**	**Genotype**	**Number of subjects**	**Frequency**
5HTTLPR	*L/L*	22	44%
	L/S	24	48%
	S/S	4	8%
5HTTLPR/rs25531	*La/La*	19	38%
	La/Lg	3	6%
	La/S	24	48%
	S/S	4	8%
STin2	*12/12*	11	22%
	12/10	31	62%
	12/9	2	4%
	10/10	6	12%
