# Supplementary material for: Epigenetic adaptation of the placental serotonin transporter gene (SLC6A4) to gestational diabetes mellitus
Source: PLoS One. 2017 Jun 26;12(6):e0179934. doi: 10.1371/journal.pone.0179934 (PMC5484502; doi:10.1371/journal.pone.0179934)
Supplement: S5 Table — (PDF) [file pone.0179934.s006.pdf]

S5 Table. Partial correlation between the *SLC6A4* methylation levels and maternal plasma glucose concentrations in the 24th to 28th week of pregnancy.

Controlling variable/s	Fasting glucose level		2 h OGTT glucose level	
	r	p-value	r	p-value
none	-0.34	**0.034**	-0.30	0.059
pBMI	-0.41	**0.009**	-0.37	**0.022**
GWG	-0.35	**0.028**	-0.34	**0.032**
pBMI, GWG	-0.41	**0.010**	-0.38	**0.018**
GA	-0.36	**0.025**	-0.28	0.086
Sex	-0.37	**0.022**	-0.29	0.071
BW	-0.36	**0.024**	-0.32	**0.048**
Genotype[a]	-0.33	**0.043**	-0.35	**0.029**
pBMI, GWG, GA, sex	-0.46	**0.004**	-0.34	**0.040**

[a]*5HTTLPR/rs25531* genotypes were grouped into *La/La* vs. other.

Number of subjects included in the analyses was 40 (OGTT results for 10 women with normal glucose tolerance status were recorded as "normal"). Statistically significant findings are shown in bold.

GA, gestational age at delivery; GWG, gestational weight gain; OGTT, oral glucose tolerance test; pBMI, pre-pregnancy body mass index; r, partial correlation coefficient.

S6 Table. Correlation of placental DNA methylation levels at individual CpG sites in the *SLC6A4* promoter region with maternal fasting plasma glucose levels in the 24th to 28th week of pregnancy, and with *SLC6A4* mRNA levels in the human placenta.

CpG [a]	Maternal fasting plasma glucose levels (n=40)		Placental *SLC6A4* mRNA relative levels (n=50)	
	r	p-value	r	p-value
4728	**-0.33** [b]	**0.035**	-0.27 [c]	0.058
4769	-0.17 [b]	0.292	-0.18 [c]	0.216
4780	-0.26 [c]	0.103	-0.20 [c]	0.157
4811	-0.25 [c]	0.113	**-0.45** [c]	**0.001**
4846	**-0.35** [c]	**0.029**	**-0.34** [c]	**0.016**
4848	**-0.38** [b]	**0.017**	**-0.38** [c]	**0.007**
4853	**-0.39** [c]	**0.013**	**-0.42** [c]	**0.003**

[a] Cytosine position according to NCBI reference sequence NG_011747.2 (GeneBank)

[b] Pearson's

[c] Spearman's

Statistically significant findings are shown in bold.

n, number of subjects; sd, standard deviation.

S7 Table. Linear regression analysis for predicting infant's birth weight (g).

**Predictor**	**B**[a]	**β**[b]	**p-value**
Gestational age (weeks)	121	0.34	0.007
Infant sex			
female	ref.		
male	217	0.26	0.039
Smoking in pregnancy			
no	ref.		
yes	- 387	- 0.41	0.002
Glucose tolerance status			
NGT	ref.		
GDM	293	0.34	0.011
GWG (kg)	18	0.29	0.029
SLC6A4 mRNA (RER)	- 474	- 0.31	0.018

[a] Unstandardized coefficient

[b] Standardized coefficient

GDM, gestational diabetes mellitus; GWG, gestational weight gain; NGT, normal glucose tolerance; RER, relative expression ratio.
