# Supplementary material for: Epigenetic adaptation of the placental serotonin transporter gene (SLC6A4) to gestational diabetes mellitus
Source: PLoS One. 2017 Jun 26;12(6):e0179934. doi: 10.1371/journal.pone.0179934 (PMC5484502; doi:10.1371/journal.pone.0179934)
Supplement: S6 Table — (PDF) [file pone.0179934.s007.pdf]

S6 Table. Correlation of placental DNA methylation levels at individual CpG sites in the *SLC6A4* promoter region with maternal fasting plasma glucose levels in the 24th to 28th week of pregnancy, and with *SLC6A4* mRNA levels in the human placenta.

CpG [a]	Maternal fasting plasma glucose levels (n=40)		Placental *SLC6A4* mRNA relative levels (n=50)	
	r	p-value	r	p-value
4728	**-0.33** [b]	**0.035**	-0.27 [c]	0.058
4769	-0.17 [b]	0.292	-0.18 [c]	0.216
4780	-0.26 [c]	0.103	-0.20 [c]	0.157
4811	-0.25 [c]	0.113	**-0.45** [c]	**0.001**
4846	**-0.35** [c]	**0.029**	**-0.34** [c]	**0.016**
4848	**-0.38** [b]	**0.017**	**-0.38** [c]	**0.007**
4853	**-0.39** [c]	**0.013**	**-0.42** [c]	**0.003**

[a] Cytosine position according to NCBI reference sequence NG_011747.2 (GeneBank)

[b] Pearson's

[c] Spearman's

Statistically significant findings are shown in bold.

n, number of subjects; sd, standard deviation.

S7 Table. Linear regression analysis for predicting infant's birth weight (g).

**Predictor**	**B[a]**	**β[b]**	**p-value**
Gestational age (weeks)	121	0.34	0.007
Infant sex			
female	ref.		
male	217	0.26	0.039
Smoking in pregnancy			
no	ref.		
yes	- 387	- 0.41	0.002
Glucose tolerance status			
NGT	ref.		
GDM	293	0.34	0.011
GWG (kg)	18	0.29	0.029
SLC6A4 mRNA (RER)	- 474	- 0.31	0.018

[a] Unstandardized coefficient

[b] Standardized coefficient

GDM, gestational diabetes mellitus; GWG, gestational weight gain; NGT, normal glucose tolerance; RER, relative expression ratio.
