# Supplementary material for: Epigenetic adaptation of the placental serotonin transporter gene (SLC6A4) to gestational diabetes mellitus
Source: PLoS One. 2017 Jun 26;12(6):e0179934. doi: 10.1371/journal.pone.0179934 (PMC5484502; doi:10.1371/journal.pone.0179934)
Supplement: S7 Table — (PDF) [file pone.0179934.s008.pdf]

S7 Table. Linear regression analysis for predicting infant's birth weight (g).

Predictor	B[a]	β[b]	p-value
Gestational age (weeks)	121	0.34	0.007
Infant sex			
female	ref.		
male	217	0.26	0.039
Smoking in pregnancy			
no	ref.		
yes	- 387	- 0.41	0.002
Glucose tolerance status			
NGT	ref.		
GDM	293	0.34	0.011
GWG (kg)	18	0.29	0.029
SLC6A4 mRNA (RER)	- 474	- 0.31	0.018

[a] Unstandardized coefficient

[b] Standardized coefficient

GDM, gestational diabetes mellitus; GWG, gestational weight gain; NGT, normal glucose tolerance; RER, relative expression ratio.
