# Supplementary material for: Epigenetic adaptation of the placental serotonin transporter gene (SLC6A4) to gestational diabetes mellitus
Source: PLoS One. 2017 Jun 26;12(6):e0179934. doi: 10.1371/journal.pone.0179934 (PMC5484502; doi:10.1371/journal.pone.0179934)
Supplement: S1 Fig — (PDF) [file pone.0179934.s009.pdf]

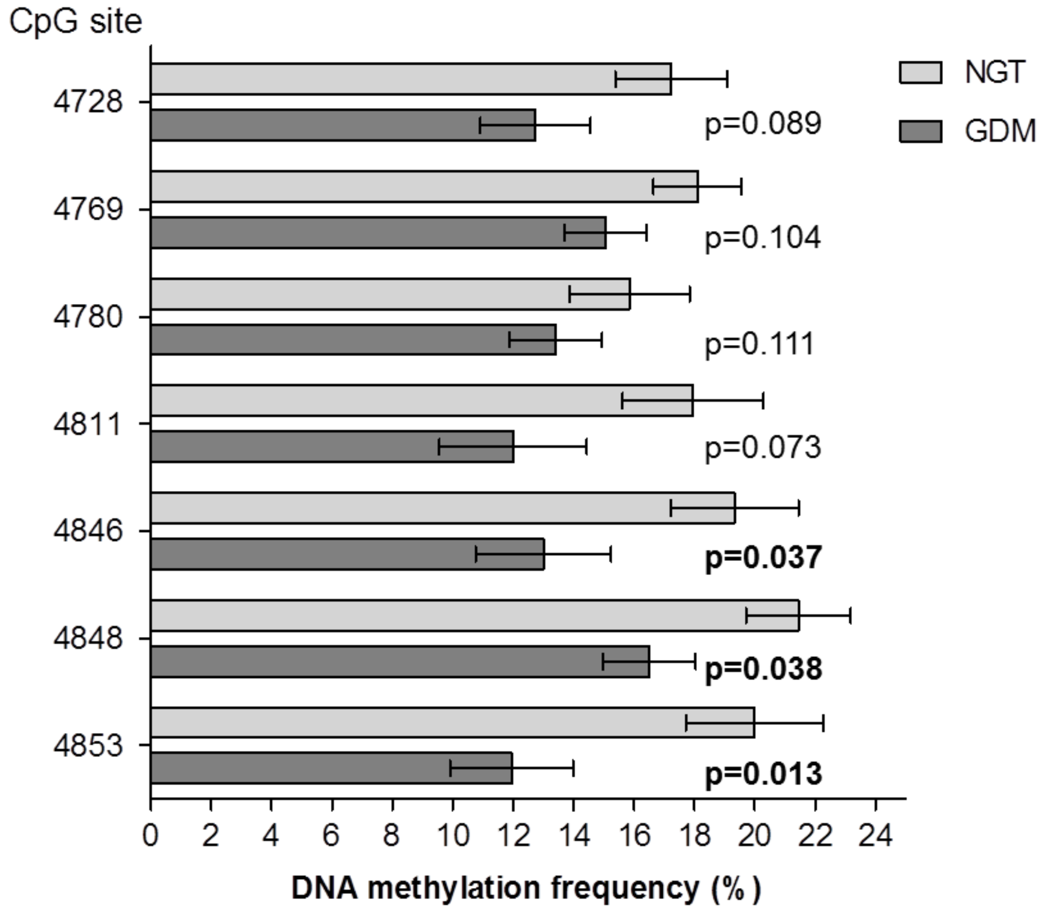

S1 Figure. Placental DNA methylation at individual *SLC6A4* loci in infants of mothers with normal glucose tolerance (NGT; n=32) and mothers with gestational diabetes mellitus (GDM, n=18). CpG sites are designated according to cytosine positions in NCBI reference sequence NG_011747.2 (GeneBank). Shown are means and standard errors. Indicated p-values were calculated by Mann-Whitney U test.
